# The effect of GnRH-a on the angiogenesis of endometriosis

**DOI:** 10.1007/s42000-024-00559-6

**Published:** 2024-04-19

**Authors:** Theodoros Filindris, Efthymia Papakonstantinou, Maria Keramida, Eleftherios Panteris, Sotiris Kalogeropoulos, Neoklis Georgopoulos, Fuminori Taniguchi, George Adonakis, Tasuku Harada, Apostolos Kaponis

**Affiliations:** 1 Dept. of Obstetrics & Gynecology, Patras University School of Medicine, Patras, Greece; 2 Laboratory of Forensic Medicine and Toxicology, School of Medicine, Aristotle University of Thessaloniki, Thessaloniki, 54124 Greece; 3 Dept. of Obstetrics & Gynecology, Tottori University Faculty of Medicine, Yonago, Japan

**Keywords:** Endometriosis, Angiogenesis, GnRH-a, VEGF, SP1

## Abstract

**Purpose:**

Neoangiogenesis is necessary for adhesion and invasiveness of endometriotic lesions in women affected by endometriosis. Vascular endothelial growth factor (VEGF) is one of the main components of angiogenesis and is part of the major pathway tissue factor (TF)-protease activated receptor-2 (PAR-2)-VEGF that leads to neoangiogenesis. Specificity protein 1 (SP1) is a transcriptional factor that has recently been studied for its crucial role in angiogenesis via a specific pathway. We hypothesize that by blocking angiogenetic pathways we can suppress endometriotic lesions. Gonadotrophin-releasing hormone-agonists (GnRH-a) are routinely used, especially preoperatively, in endometriosis. It would be of great interest to clarify which angiogenetic pathways are affected and, thereby, pave the way for further research into antiangiogenetic effects on endometriosis.

**Methods:**

We used quantitative real-time polymerase chain reaction (qRT-PCR) to study mRNA expression levels of TF, PAR-2, VEGF, and SP1 in endometriotic tissues of women who underwent surgery for endometriosis and received GnRH-a (leuprolide acetate) preoperatively.

**Results:**

VEGF, TF, and PAR-2 expression is significantly lower in patients who received treatment (*p* < 0,001) compared to those who did not, whereas SP1 expression is not altered (*p* = 0.779).

**Conclusions:**

GnRH-a administration does affect some pathways of angiogenesis in endometriotic lesions, but not all of them. Therefore, supplementary treatments that affect the SP1 pathway of angiogenesis should be developed to enhance the antiangiogenetic effect of GnRH-a in patients with endometriosis.

**Trial registration:**

Clinicaltrial.gov ID: NCT06106932.

**Supplementary Information:**

The online version contains supplementary material available at 10.1007/s42000-024-00559-6.

## Introduction

Endometriosis is a chronic benign gynecological disease that is characterized by the presence of endometriotic tissue outside the uterine cavity [1]. It is an estrogen-dependent inflammatory disease that affects up to 5 to 10% of reproductive-aged women and is associated with pain and/or infertility among 30 to 50% of these women [1]. Retrograde menstruation and peritoneal adhesion of endometrial tissue are basic elements in the pathogenesis of endometriosis according to Sampson’s classical implantation theory [2]. However, what causes attachment and outgrowth of endometrial cells after their appearance in the peritoneal cavity remains unclear.

Similarly to tumors and metastases, endometriotic lesions require development of new blood vessels for continuous oxygen and nutrient supply [3]. Endometriotic lesions produce cytokines and growth factors that promote their proliferation and vascularization [4]. Interleukin-1β (IL-1β), IL-6, and IL-8 are cytokines that have a stimulatory role in angiogenesis and neovascularization in endometriotic lesions [5–7]. Many angiogenic growth factors have been shown to be overexpressed in endometriotic lesions and in the peritoneal fluid of women with endometriosis [8, 9]. Τhere is increasing evidence that the vascular endothelial growth factor (VEGF) family is involved in the etiology and maintenance of peritoneal endometriosis [9]. 17β-estradiol (E2) up regulates VEGF expression in human endometrial stromal cells [10, 11].

Tissue factor (TF) is also known to be involved in angiogenesis via intracellular signaling that utilizes protease activated receptor-2 (PAR-2), as indicated in multiple studies [12, 13]. Specificity protein 1 (SP1) is thought to regulate VEGF expression in several carcinomas, such as pancreatic adenocarcinoma and ovarian cancer [14, 15].

Gonadotropin-releasing hormone agonists (GnRH-a) have long been used for the management of endometriosis. The administration of long-lasting GnRH agonists has a central effect, causing pituitary down-regulation and a reduction in gonadotropin release, thus exerting an impact on endometriotic lesions [16]. According to the latest ESHRE guideline, it is strongly recommended that GnRH-a be prescribed in patients to reduce endometriosis-associated pain [17]. Considering the significance of angiogenesis in endometriotic lesions, it has been hypothesized that GnRH-a might influence angiogenic mechanisms in endometrial cell growth [16]. According to a published study, GnRH-a (leuprolide acetate, LA) has a direct effect on endometriotic tissue, partly by interfering in inhibiting angiogenesis [18]. There is, hence, great interest in utilizing this knowledge since it may pave the way to further investigation regarding the effect of GnRH analogs on angiogenesis in endometriotic lesions.

In the current study, we explore the effect of the long-term administration of a GnRH-a on angiogenesis factors which promote neoangiogenesis via two different and independent pathways, namely, the TF-PAR-2-VEGF pathway and the SP-1-VEGF pathway.

## Materials & methods

The subjects in this study were women of reproductive age. From January 2016 to December 2022, 60 women with known endometriosis (stages 2 and 3) were recruited. The staging of endometriosis was based on the rASRM classification system [19]. Stage 2 includes women with ovarian endometrioma and superficial ovarian endometriosis, peritoneal filmy adhesions, or deep peritoneal endometriosis. Stage 3 includes women with ovarian endometrioma, deep peritoneal endometriosis with dense adhesions, and partial obliteration of the cul de sac. Their mean age was 38 years. They were nulliparous and had a mean body mass index (BMI) of 27 kg/m^2^. The ovarian endometrioma present in all the participants was diagnosed using ultrasonography and/or magnetic resonance imaging. Women with perimenopausal symptoms such as hot flashes, night sweats, and/or irregular menstrual period were excluded from the current study. The inclusion and exclusion criteria are presented in Table 1.

**Table 1:** Inclusion and exclusion criteria of women

Inclusion criteria	Exclusion criteria
Reproductive age	Perimenopausal symptoms *(hot flashes, night sweats, irregular menstrual bleeding)*
Personal history of infertility	FSH > 12 IU/dl*
Endometriosis stage II or greater	Obesity**
BMI < 35 kg/m2	Hormonal treatment 1 year before the evaluation
Endometriomas > 5 cm	

*Measured on 2^nd^ day of menstrual period. **BMI>35 kg/m2.

This was a randomized follow-up study with analysis of ovarian samples derived from GnRH-a-treated and non-GnRH-a-treated women before surgery. The randomization was performed by accessing a central internet-based randomization program MinimRan [20]. The random allocation sequence and the assignment of the participants to interventions were made by two of the authors (A.K. and S.K).

After enrollment, the women were randomized into two groups (Table 2). Group A (GnRHa+) consisted of 30 women with a mean age of 35.5 years and a mean BMI of 27 kg/m^2^. Seventeen of them had stage 2 and 13 had stage 3 endometriosis. They received GnRH-a (LA) for a period of 3 months prior to surgery and had not received any hormonal treatment within the 12 months before the surgical procedure. Group B (GnRHa-) consisted of 30 women with a mean age of 38 years and a mean BMI of 27 kg/m^2^. Sixteen of them had stage 2 and 14 had stage 3 endometriosis. They did not receive GnRH-a treatment before surgery. In addition, no treatment with oral contraceptives or other hormonal therapy had been administered within 12 months prior to surgery.

**Table 2:** Demographic parameters of the study population and controls. Stage of endometriosis

No. of participants	30	30	-
	Group A	Group B	-
Age [median, 95% Cis]	35.5 [29–40]	38.00 [34–46]	0.388
BMI [median, 95% Cis]	27.00	27.00	0.910
Duration of Treatment [months]	3	-	-
Stage 2 endometriosis	17	16	0.924
Stage 3 endometriosis	13	14	0.956
Menstrual cycle phase	Amenorrhea	Proliferative	-

During laparoscopy, biopsy specimens of the ovarian endometrioma were collected. In group B, surgery was performed during the proliferative phase of the menstrual cycle. All biopsy specimens were collected in accordance with the guidelines of the Declaration of Helsinki and with the approval of the ethical committee of the General University Hospital of Patras. Informed consent was obtained from all women.

### qRT-PCR

Quantitative real-time polymerase chain reaction (qRT-PCR) is used to study the expression of genes in various tissues. This method is one of the most common tools, enabling relative quantification of target gene expression by comparison with the expression of a “reference” or “housekeeping” gene. A “housekeeping” gene is defined as being constitutively expressed in the tissue under study [21]. The reference gene should have stable expression under all experimental conditions (i.e., patients and controls) and be expressed appropriately in the tissue studied, otherwise results may be biased.

### For primer design

The gene sequences of the exons and introns used for the design of the specific primers were obtained from the ensembl database (EMBL-EBI) (Online Resource 1). Primer design, purchased from Thermo Fisher Scientific, using the gene sequences was performed with the NCBI tool “Primer-Blast” according to the instructions of the manufacturer. The following criteria were considered in the development of the primers [22]:

1. Length of 18–24 bases
2. 40–60% G/C content
3. Starts and ends with 1–2 G/C pairs
4. Melting temperature (TM) of 50–65 °C
5. The two primers of a primer pair should have closely matched melting temperatures for maximizing PCR product yield
6. Primer pairs should not have complementary regions
7. The amplicon length is dictated by the experimental goals. For qPCR, the target length is closer to 100 bp and for standard PCR it is near 500 bp (Online Resource 2; Online Resource 3)

Fresh tissue samples were cut < 0.5 cm and immersed in 5–10 volumes of RNAlater Stabilization Solution (Invitrogen, Cat. No. AM7020), stored at 4 °C overnight, and then moved to − 80 °C until RNA extraction for long-term storage.

### Tissue lysis and RNA extraction

Prior to RNA isolation, the samples were lysed and homogenized. The frozen tissue was placed on ice and 0.5 mL of TRIzol Reagent (TRIzol Reagent, Cat. No: 15,596,026, Invitrogene) was added at optimal sample size (50–70 mg) and homogenized at 25 Hz for 3 min. 0.1 mL of chloroform was added to 0.5 mL of Trizol reagent, shaken vigorously by hand for 15 s, and incubated at room temperature for 3 min. The samples were centrifuged at 11.600 x g for 15 min at 4 °C. An equal volume of ice-cold 75% ethanol was added to the upper phase and transferred to a High Pure Filter Tube of the High Pure RNA Isolation Kit (Cat. No. 11 828 665 001, Roche) [23]. RNA isolation was performed according to the isolation kit protocol [24]. The concentration and purity of RNA was determined by measuring the absorbance at 260 nm and 280 nm in a spectrophotometer. The yield of total RNA was 0.5–0.8 µg/mg.

DNA (cDNA) synthesis was performed with a mixture of anchored-oligo (dT) primers and 1 µg of total RNA, according to the manufacturer’s instructions (Transcriptor First Strand cDNA Synthesis Kit, Cat. No. 04897030001; Roche Applied Science). Real-time PCR was carried out in the LightCycler 2 Instrument (Roche) using the FastStart Universal SYBR Green Master (Roche Hellas).

Four independent experiments were analyzed in duplicates for all data shown. GAPDH was used as a reference gene for normalization. To analyze qPCR data, REST-MCS beta software version 2 was used.

### Statistical methods

Power analysis was performed using GPower 3.1.9.6 for the comparison of patients with and without GnRH-a for a power level of = 0.8 with effect size = 0.7. Effect size was deemed to be large (0.7), as a large difference is expected between RNA expression of the main regulators of angiogenesis between patients with and without GnRH-a [25]. A sample size of 28 per group was indicated for the specified power level; thus, 60 patients were recruited to allow for dropouts/analysis issues.

The data were analyzed using nonparametric methods via SPSS (Statistical Package for the Social Sciences) v. 26 (SPSS, Inc. Chicago, IL, USA) to generate graphs and analyses. As parameters do not follow the normal distribution as per the Shapiro-Wilk normality test, the Mann-Whitney U test was used for multiple variables with a significance level of 0.05. Median and 95% confidence intervals (95% CIs) were recorded for all continues variables.

## Results

A sample of 60 women, 30 treated with GnRH-a in the amenorrhea phase and 30 controls without GnRH-a treatment in the proliferative menstrual cycle phase participated in this study. The stage of endometriosis was similar in both groups (*p* = 0.956). There were no demographic differences among the patients as age and BMI were similar in the two groups, with a median age of 38 years old (34–46, 95%CIs) for the control group, who did not receive GnRH-a antagonists, and a median age of 35.5 years old (30–41, 95%CIs) (*p* = 0.388) for the experimental group, who did. The BMI was also identical between the two groups (*p* = 0.910). Table 2 displays the demographics.

**Fig. 1:**
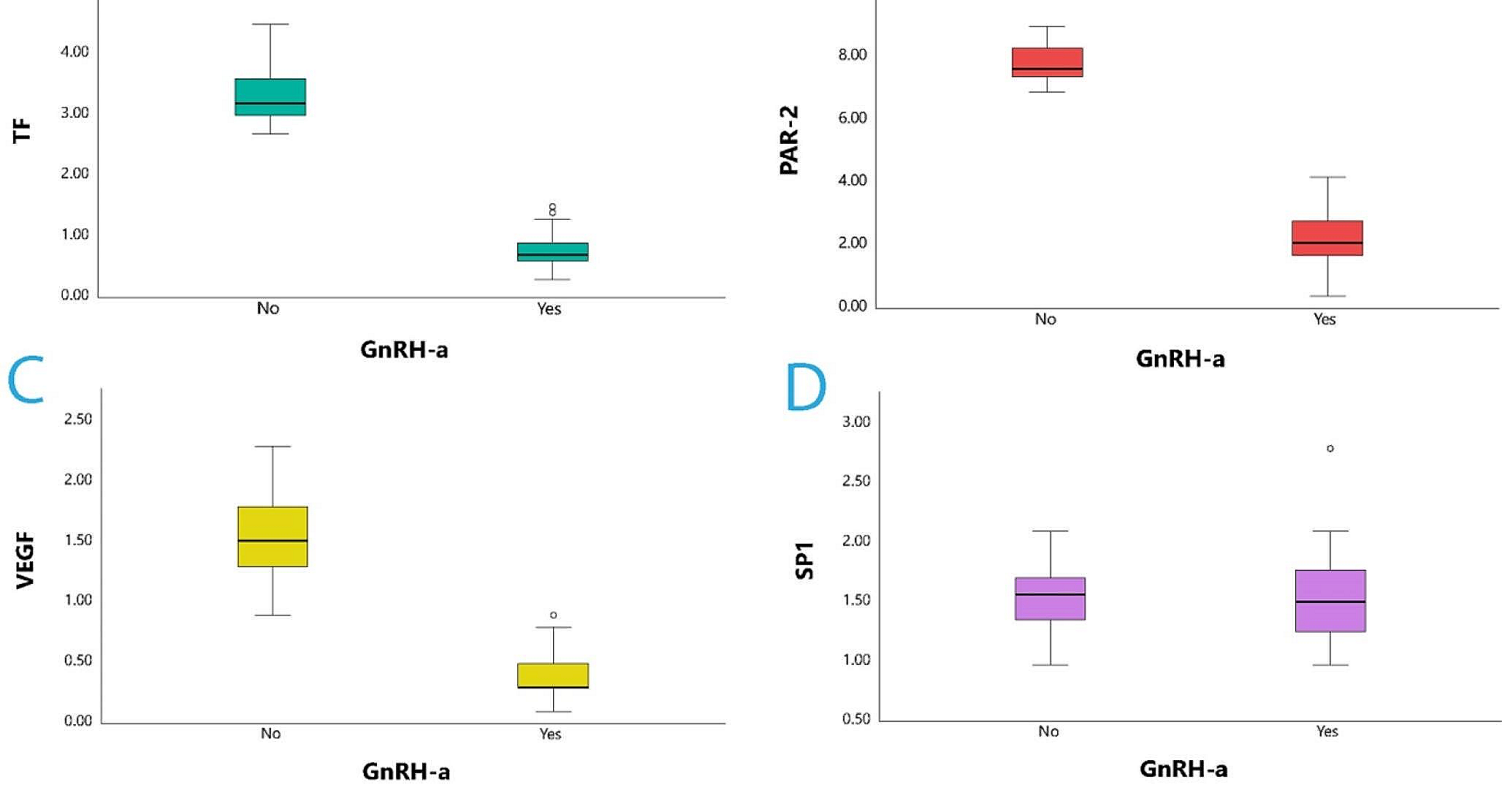
Gene expression of PAR-2, TF, VEGF, and SP-1 in endometriotic tissues of women with and without GnRH-a administration

Median expressions of the relevant mRNAs were statistically differentiated between the two groups. In detail, *TF* mRNA median expression was 3.2 (3.1–3.6, 95%CIs) in control vs. 0.7 (0.7–0.9, 95%CIs) in the experimental group. Similarly, *PAR-2* mRNA median expression was 7.65 (7.5–8.2, 95%CIs) vs. 2.1 (1.9–2.7, 95%CIs) in the control versus the experimental group and *VEGF* mRNA median expression was also significantly differentiated with 1.52 (1.4–1.8 95%CIs) vs. 0.3 (0.3–0.4, 95%CIs) in the control versus the experimental group, respectively. In contrast, *SP1* mRNA did not show any differentiation between the two groups, with *SP1* median expression being 1.57 (1.43–1.69, 95%CIs) vs. 1.51 (1.36–1.69, 95%CIs) in the control versus the experimental group, respectively. Table 3; Fig. 1 presents the latter specifics in detail.

**Table 3:** Gene expression between women with endometriosis who received a GnRH-a (Group A) and those who did not receive GnRH-a (Group B)

	Group A		Group B		Mann -Whitney U test
	Median	95.0% CΙs	Median	95.0% CΙs	*P* value
TF	0.7	0.70–0.90	3.2	3.10–3.60	< 0.001
PAR-2	2.1	1.90–2.70	7.65	7.50–8.20	< 0.001
VEGF	0.3	0.30–0.40	1.52	1.40–1.80	< 0.001
SP1	1.51	1.36–1.69	1.57	1.43–1.69	0.779

## Discussion

In normal endometrial stromal cells, VEGF is highly expressed, its levels depending on the effect of estrogen and progesterone [26–28]. It is widely accepted that in women with endometriosis, VEGF is highly expressed in peritoneal fluid as well as in ectopic endometrial tissue [9, 26, 29, 30]. Estrogen is a proangiogenic hormone whose effects on neovascularization and angiogenesis in the uterus and endometrium through proliferation and migration of endothelial cells are widely studied [26]. Previous studies have reached the conclusion that GnRH-a (LA) administration in women with endometriosis or uterine fibroids downregulated VEGF expression and affected the vascular pattern via decrease of microvessel density in the endometria studied [31–33]. The above observations enable us to formulate the hypothesis that reduction in the size of endometriomas after treatment with GnRH-a might be caused by reduction of angiogenesis in the pathologic lesions.

Many other components apart from VEGF are involved in neoangiogenesis and thus play an equally significant role in this pathway. Angiogenesis has been widely studied in neoplastic tissues given that angiogenesis is of great importance for tumor viability and progression [34, 35]. TF is a cell membrane-bound glycoprotein that binds to circulating factor VIIa to mediate the activation of both factors IX and X and, thus, has a crucial role in hemostasis [35]. According to a number of studies, TF-PAR2 signaling contributes to angiogenesis. When TF is exposed to the bloodstream, FVIIa binds to it on the cell surface, an event that promotes hemostasis. Furthermore, the binding of FVIIa to TF cleaves PAR-2, a cleavage that results in phosphorylation of the TF cytoplasmic domain and inhibits the negative effect of PAR-2-mediated signaling, promoting angiogenesis [35]. Several mitogen-activated protein kinase (MAPK) pathways are then activated, which leads to expression of several genes, one of them being the VEGF gene. High expression of VEGF has been reported after exposure of TF-expressing cells to FVIIa (via PAR-2 activation). Our observation that mRNA of TF and PAR-2 is downregulated in women receiving GnRH-a is significant as regards insight into the role of GnRH-a in angiogenesis, since it blocks one of the most important pathways, thereby causing endometriosis regression.

Transcription factor SP1 promotes tumor angiogenesis and invasion by activating VEGF expression in several tumors, such as ovarian and pancreatic cancer, following a different and independent pathway from that of TF-PAR2-VEGF [14, 35]. According to a recent study, SP1 can activate the transforming growth factor-β1/Sma and Mad proteins 2 (TGF-β1/SMAD2) pathway and promote VEGF secretion through TGF-β1, promoting angiogenesis in preosteoblasts [37]. In gastric cancer as well, transcription factor SP1 is an independent prognostic factor since it has been observed that the higher the expression of SP1, the higher the microvessel density (MVD) of the tumor [38]. In a recent study, SP1 mRNA and protein levels were found to be increased in ectopic and eutopic endometrium of women with stage III/IV endometriosis [39]. We therefore included SP1 transcription factor in our study and found that GnRH-a does not affect its expression in endometriomas. The non-involvement of SP1 could be due to its known post-translational modification capacity, which regulates its expression, which action could override potential disruptors. SP1 is a unique transcription factor as it both initiates transcription and can regulate the activation and repression processes: it is thus a key component that must not be affected by external disruptors [40, 41].

Hormonal treatments, such as GnRH-a, are not suitable for women desiring to preserve their fertility and act only for symptomatic relief and not for actual improvement of fertility [42]. Bevacizumab, an anti-VEGF, non-hormonal factor has been studied for possible treatment of endometriosis; however, it carries serious, not easily tolerated side effects (i.e., gastrointestinal perforation, thrombosis, severe bleeding, impaired kidney function, and wound healing) [42]. In addition, statins work in a dose-dependent way, either promoting angiogenesis (at low doses) or blocking angiogenesis (at higher doses). However, their long-term side effects, such as myopathy and rhabdomyolysis, as well as their controversial effectiveness remain a deterrent factor to their widespread use [42]. Cabergoline, a dopamine agonist, has also been studied for the treatment of endometriosis and, in some studies, it has been found to downregulate VEGF receptors in endometriotic implants [42, 43]. Future research is essential to highlight the role of other treatments, hormonal or non-hormonal, in downregulating both the TF-PAR2-VEGF and SP1 pathways of angiogenesis, resulting in ultimately diminishing endometrioma size and not only relieving the symptoms.

Recent studies on the treatment of endometriosis have focused on the development of antiangiogenic drugs, such as anti-VEGF antibodies, VEGFR tyrosine kinase, COX-2 inhibitors, and dopamine agonists [44]. This is why the present study is of high originality, given the fact that it is the first time, to our knowledge, that a study has explored the impact of GnRH-a treatment preoperatively on angiogenetic pathways in women with endometriosis. One disadvantage of the present study is that we studied only the mRNA expression of TF, PAR2, VEGF, and SP1 and not their protein expression using Western blot or enzyme-linked immunosorbent assay (ELISA). While GnRH-a can inhibit neoangiogenesis in endometriotic lesions, it cannot completely block all the angiogenetic pathways, since no alterations in expression of the SP1 pathway of angiogenesis have been found. Further research should be conducted to discover new, more efficient treatments of endometriosis. Since endometriosis concerns many reproductive-aged women, discovering ways to affect its angiogenesis is very promising to moderate the role of angiogenesis in its pathogenesis and will give hope and new perspectives to patients with endometriosis.

## Electronic supplementary material

Below is the link to the electronic supplementary material.

Supplementary Material 1

Supplementary Material 2

## Abbreviations

BMI: Body mass index
ELISA: Enzyme-linked immunosorbent assay
E2: 17β-estradiol
GnRH-a: Gonadotropin-releasing hormone agonist
IL-1β: Interleukin 1β
MVD: Microvessel density
MAPK: Mitogen activated protein kinase
PAR-2: Protease activated receptor 2
qRT-PCR: Quantitative real-time polymerase chain reaction
SP1: Specificity protein 1
SMAD2: Sma and Mad proteins from *Caenorhabditis elegans* and *Drosophilla*, respectively
TF: Tissue factor
TGF-β1: Transforming growth factor β1
VEGF: Vascular endothelial growth factor
