# Supplementary material for: The effect of GnRH-a on the angiogenesis of endometriosis
Source: Hormones (Athens). 2024 Apr 19;23(3):509–15. doi: 10.1007/s42000-024-00559-6 (PMC11436414; doi:10.1007/s42000-024-00559-6)
Supplement: Supplementary file 2 — Supplementary Material 2 [file 42000_2024_559_MOESM2_ESM.pdf]

Online Resource 1. Genes Sequences Transcript ID

Genes Sequences	Transcript ID
Sp1 gene [Sp1 transcription factor]	ENST00000327443.9
VEGFA gene [vascular endothelium growth factor A]	ENST00000372055.9
PAR-2 gene/F2RL1 [F2R like trypsin receptor 1]	ENST00000296677.5
F3 gene/coagulation factor III/tissue factor [TF]	ENST00000334047.12

Online Resource 2. Oligonucleotide primers used in quantitative RT-PCR [real-time polymerase chain reaction]

Gene	Forward primer	Reverse Primer
Sp1	5'-GACCCCCTTGAGCTTGTCCCT-3' [21b]	5'-CTGTGAAAAGGCACCACCACC-3' [21b]
VEGFA	5'-TTGCCTTGCTGCTCTACCTCCA-3' [22b]	5'-GATGGCAGTAGCTGCGCTGATA-3' [22b]
PAR-2	5'-CTGAGTTTCGAATCGGCGG-3' [19b]	5'-GGATGTGCCATCAACCTTACC-3' [21b]
TF	5'-AGACAGCCCGGTAGAGTGTATG-3' [22b]	5'-TGCCCCACTCCTGCCTTTCTAC-3' [22b]
GAPDH	5'-CCATGTTCGTCATGGGTGTGA-3' [21b]	5'CATGGACTGTGGTCATGAGT-3' [20b]

Online Resource 3. Tm of selected primers

Primer	Tm [Melting Temperature]
SP1F	63,81
SP1R	61,96
VEGFAF	63,93
VEGFAR	62,79
PAR2F	59
PAR2R	58,98
TFF	61
TFR	64
GAPDHF	60,61
GAPDHR	57,22
