# Supplementary material for: The effect of GnRH-a on the angiogenesis of endometriosis
Source: Hormones (Athens). 2024 Apr 19;23(3):509–15. doi: 10.1007/s42000-024-00559-6 (PMC11436414; doi:10.1007/s42000-024-00559-6)
Supplement: Supplementary file 1 — Supplementary Material 1 [file 42000_2024_559_MOESM1_ESM.docx]

Patra 2024/04/03

For the Editor in Chief

Hormones-International Journal of Endocrinology and Metabolism

Constantine A. Stratakis

Dear Editor-in-Chief

The authors of the current study want to thank you very much for giving us the opportunity to publish at the excellent Hormones Journal. We would like also to thank you very much for the language grammatical correction of the current manuscript.

We hope and wish for another fruitful collaboration in the near future.

Respectfully

Apostolos Kaponis

Asoc. Professor of Obstetrics & Gynecology

Patras University School of Medicine
